# Supplementary material for: The complete mitochondrial genome of Calyptogena marissinica (Heterodonta: Veneroida: Vesicomyidae): Insight into the deep-sea adaptive evolution of vesicomyids
Source: PLoS One. 2019 Sep 19;14(9):e0217952. doi: 10.1371/journal.pone.0217952 (PMC6752807; doi:10.1371/journal.pone.0217952)
Supplement: S2 Table — (DOCX) [file pone.0217952.s002.docx]

**S2 Table. Species used for CodeML analyses of selective pressure on mitochondrial genes.**

| Species | Subclass | Order | Superfamily | Family | GenBank  Accession No. | Genome  Length(bp) |  |
| --- | --- | --- | --- | --- | --- | --- | --- |
| *Loripes lacteus* | Heterodonta | Lucinida | Lucinoidea | Lucinidae | EF043341 | 17321 |  |
| *Lucinella divaricata* | Heterodonta | Lucinida | Lucinoidea | Lucinidae | EF043342 | 18940 |  |
| *Panopea generosa* | Heterodonta | Myoida | Hiatelloidea | Hiatellidae | NC_025635 | 15585 |  |
| *Panopea globosa* | Heterodonta | Myoida | Hiatelloidea | Hiatellidae | NC_025636 | 15469 |  |
| *Acanthocardia tuberculata* | Heterodonta | Veneroida | Cardioidea | Cardiidae | DQ632743 | 16104 |  |
| *Cerastoderma edule* | Heterodonta | Veneroida | Cardioidea | Cardiidae | NC_035728 | 14947 |  |
| *Donax semiestriatus* | Heterodonta | Veneroida | Cardioidea | Donacidae | NC_035984 | 17044 |  |
| *Donax vittatus* | Heterodonta | Veneroida | Cardioidea | Donacidae | NC_035987 | 17070 |  |
| *Soletellina diphos* | Heterodonta | Veneroida | Tellinoidea | Psammobiidae | NC_018372 | 16352 |  |
| *Semele scabra* | Heterodonta | Veneroida | Tellinoidea; | Semelidae | NC_018374 | 17117 |  |
| *Solecurtus divaricatus* | Heterodonta | Veneroida | Tellinoidea | Solecurtidae | NC_018376 | 16749 |  |
| *Moerella iridescens* | Heterodonta | Veneroida | Tellinoidea; | Tellinidae | NC_018371 | 16799 |  |
| *Solen grandis* | Heterodonta | Veneroida | Solenoidea | Solenidae | NC_016665 | 16784 |  |
| *Solen strictus* | Heterodonta | Veneroida | Solenoidea | Solenidae | NC_017616 | 16535 |  |
| *Coelomactra antiquata* | Heterodonta | Veneroida | Mactroidea | Mactridae | JN692486 | 17384 |  |
| *Mactra chinensis* | Heterodonta | Veneroida | Mactroidea | Mactridae | KJ754823 | 17285 |  |
| *Lutraria maxima* | Heterodonta | Veneroida | Mactroidea | Mactridae | NC_036766 | 17082 |  |
| *Lutraria rhynchaena* | Heterodonta | Veneroida | Mactroidea | Mactridae | NC_023384 | 16927 |  |
| *Abyssogena mariana* | Heterodonta | Veneroida | Glossoidea | Vesicomyidae | LC126311 | 15927^♣^ |  |
| *Abyssogena phaseoliformis* | Heterodonta | Veneroida | Glossoidea | Vesicomyidae | AP014557 | 19424 |  |
| *Archivesica pacifica* | Heterodonta | Veneroida | Glossoidea | Vesicomyidae | MF959624 | 17782^♣^ |  |
| *Archivesica gigas* | Heterodonta | Veneroida | Glossoidea | Vesicomyidae | MF959623 | 15674^♣^ |  |
| *Archivesica sp.* | Heterodonta | Veneroida | Glossoidea | Vesicomyidae | MF959622 | 15650^♣^ |  |
| *Calyptogena* *magnifica* | Heterodonta | Veneroida | Glossoidea | Vesicomyidae | NC_028724 | 19738 |  |
| *Calyptogena marissinica* | Heterodonta | Veneroida | Glossoidea | Vesicomyidae |  | 17374 |  |
| *Isorropodon fossajaponicum* | Heterodonta | Veneroida | Glossoidea | Vesicomyidae | AP014550 | 19556^♣^ |  |
| *Phreagena okutanii* | Heterodonta | Veneroida | Glossoidea | Vesicomyidae | AP014555 | 16336^♣^ |  |
| *Meretrix lusoria* | Heterodonta | Veneroida | Veneroidea | Veneridae | GQ903339 | 20268 |  |
| *Meretrix meretrix* | Heterodonta | Veneroida | Veneroidea | Veneridae | GQ463598 | 19826 |  |
| *Paphia textile* | Heterodonta | Veneroida | Veneroidea | Veneridae | NC_016890 | 18561 |  |
| *Paphia undulata* | Heterodonta | Veneroida | Veneroidea | Veneridae | NC_016891 | 18154 |  |
| *Dosinia japonica* | Heterodonta | Veneroida | Veneroidea | Veneridae | NC_038063 | 17693 |  |
| *Dosinia troscheli* | Heterodonta | Veneroida | Veneroidea | Veneridae | NC_037917 | 17229 |  |
| *Chlamys farreri* | Pteriomorphia | Pectinida | Pectinoidea | Pectinidae | NC_012138 | 21695 |  |
| *Mimachlamys nobilis* | Pteriomorphia | Pectinida | Pectinoidea | Pectinidae | FJ415225 | 17963 |  |

Note: ^♣^ indicated incomplete mitogenome with the control region and/or some tRNA genes missing.
