# Supplementary figures and images for: The complete mitochondrial genome of Calyptogena marissinica (Heterodonta: Veneroida: Vesicomyidae): Insight into the deep-sea adaptive evolution of vesicomyids
Source: PLoS One. 2019 Sep 19;14(9):e0217952. doi: 10.1371/journal.pone.0217952 (PMC6752807; doi:10.1371/journal.pone.0217952)

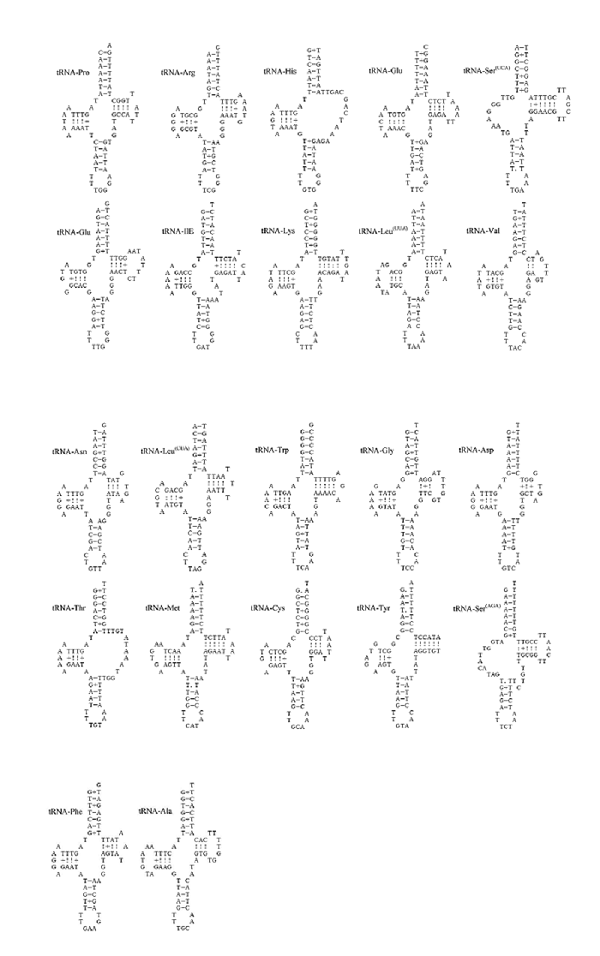

Supplement: S1 Fig — (TIF) [file pone.0217952.s003.tif]

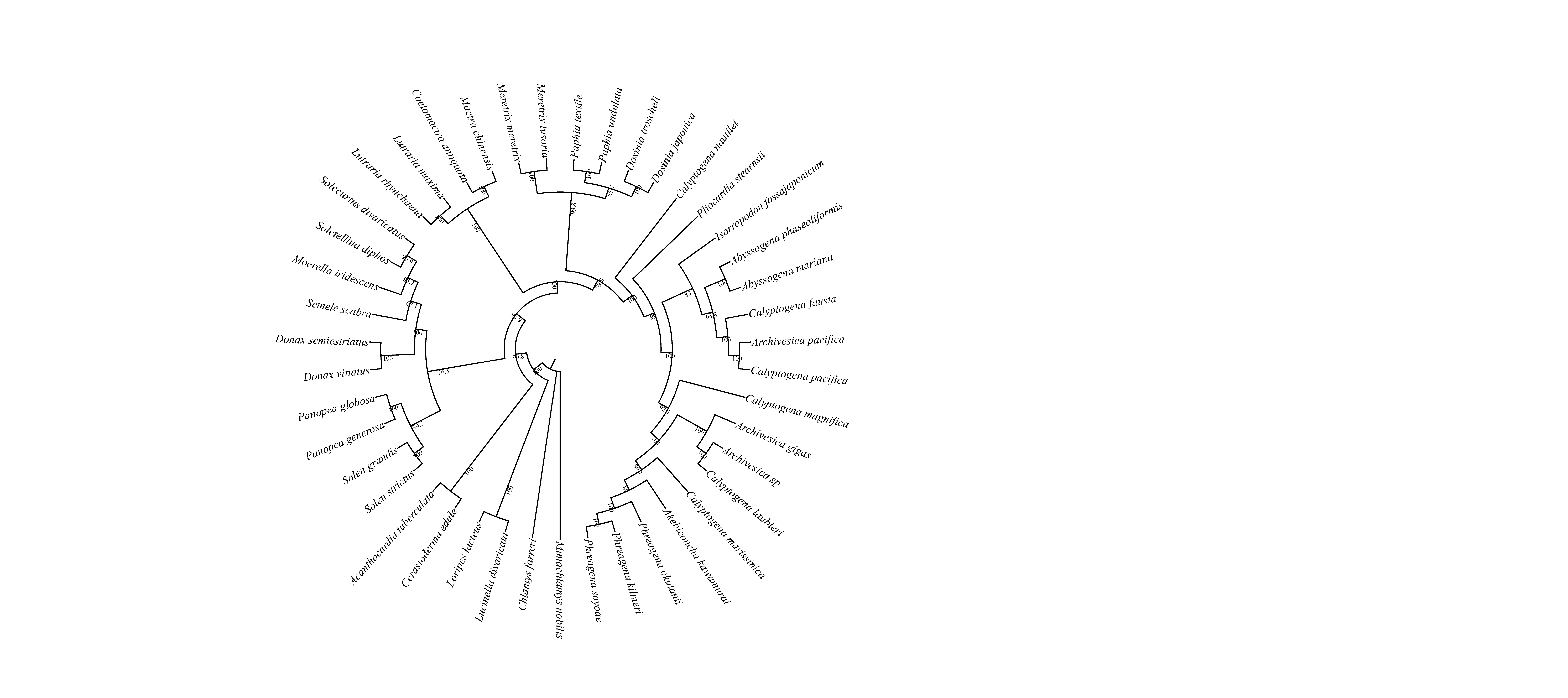

Supplement: S2 Fig — (TIF) [file pone.0217952.s004.tif]

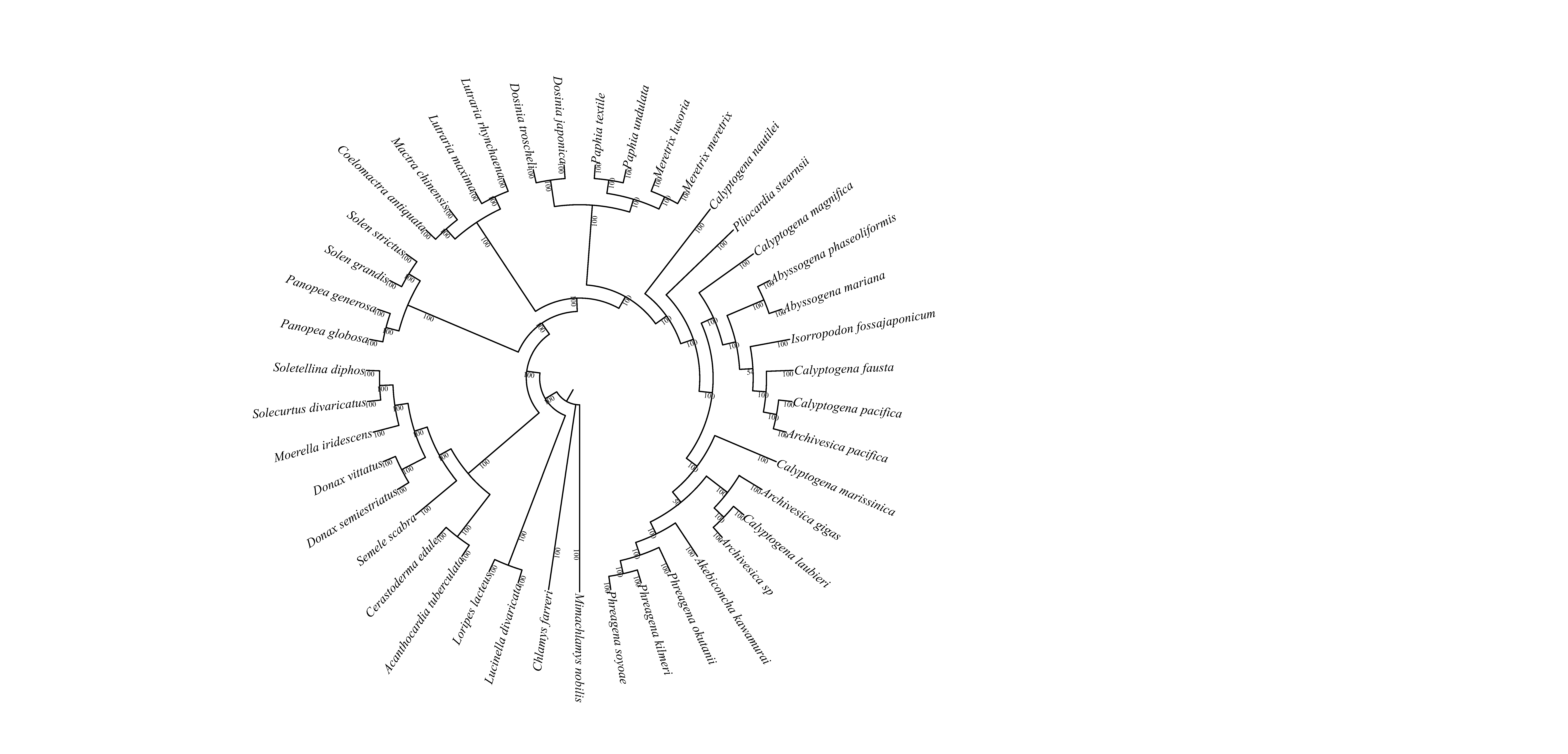

Supplement: S3 Fig — (TIF) [file pone.0217952.s005.tif]

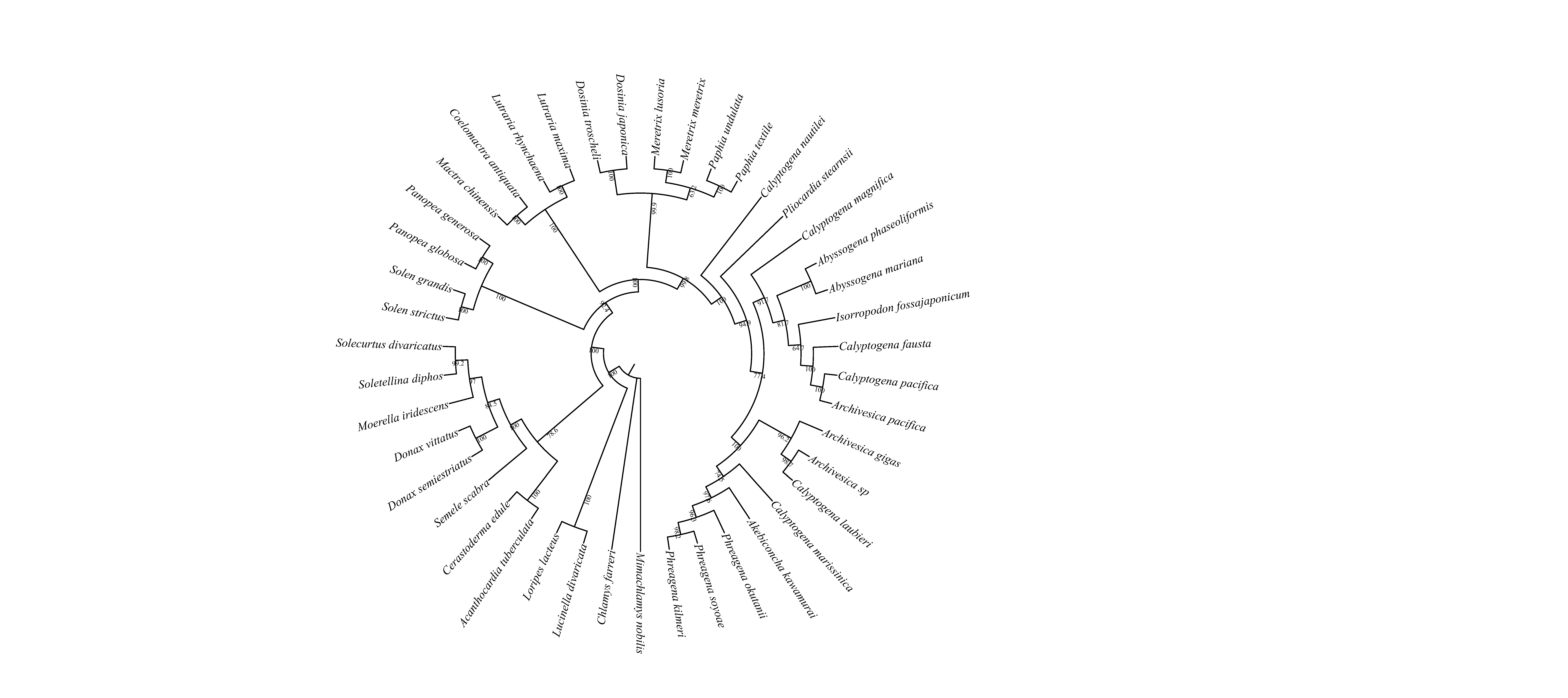

Supplement: S4 Fig — (TIF) [file pone.0217952.s006.tif]
